# Broadening bandgaps in a multi-resonant piezoelectric metamaterial plate via bandgap merging phenomena

**DOI:** 10.1038/s41598-024-66849-6

**Published:** 2024-07-12

**Authors:** Yuhao Li, Zhiyuan Liu, Hao Zhou, Kaijun Yi, Rui Zhu

**Affiliations:** School of Aerospace Engineering, Beijing Institute of Technology, Beijing, China

**Keywords:** Metamaterial, Piezoelectric material, Parameter optimization, Bandgap, Elastic wave isolation, Mechanical engineering, Structural materials

## Abstract

Locally resonant metamaterials usually have narrow bandgaps, which significantly limits their applications in realistic engineering environments. In this paper, an optimization method based on the genetic algorithm is proposed to broaden bandgaps in multi-resonant piezoelectric metamaterial through the merging of multiple separated bandgaps. Using the effective medium theory, the equivalent bending stiffness and dispersion relationship of a metamaterial plate are first obtained. Then, the criteria for determining the bandgap ranges for the two cases with and without damping are provided and analyzed. Furthermore, based on the bandgap merging phenomena, an optimization method for widening the bandgap is proposed based on the genetic algorithm. By investigating the bandgap widening effects in cases without and with damping, it is found that, when there is no damping, the bandgap can only be slightly widened; while after introducing damping into the transfer functions, the bandgap can be significantly widened by more than 200%. The bandgap widening effects are verified by comparing with finite element simulation results.

## Introduction

Plates and shells are widely used as load-bearing structures in various equipment like carrier rockets and underwater vessels. These structures not only need to meet static the load requirements but also require satisfying vibration and acoustic performance. Since structural vibration and the resulting noise radiation are closely related to elastic wave propagation in the structure, isolating the propagation of elastic waves from the source to the target parts of the structure proves to be an effective approach for vibration reduction and noise control. The concept of elastic metamaterials with local resonators provides a new approach to deal with elastic waves. Elastic metamaterials can generate low-frequency bandgaps with subwavelength units, which effectively block the propagation of elastic waves within the desired bandgap frequency range. However, most metamaterials have narrow bandgaps, severely limiting the practical application of elastic metamaterials in real-world engineering applications.

Various approaches have been proposed to broaden the frequency range of metamaterials’ bandgaps. Among them, the approach of adding multiple resonators to rod^1^, beam^2^, and plate^3^ structures is often utilized. By increasing the number of carefully tuned resonators in the unit cell, a wider bandgap can be achieved in the multi-resonant metamaterial^4^. For instance, Zhu et al.^2^ introduced an elastic metamaterial beam with multiple embedded local resonators to achieve broadband vibration suppression without sacrificing its load-bearing capacity. Additionally, widening the bandgap can be achieved through the design of gradient structures^5^. For example, Lewinska et al.^6^ proposed and investigated a gradient acoustic metamaterial where a distribution of local resonators with different characteristics (mass and stiffness) was introduced. The results indicate that by properly choosing the mass and stiffness of the distributed resonant unit cells, the frequency attenuation range can be effectively expanded. Furthermore, broad coupling bandgaps can be formed by coupling localized resonance bandgaps with Bragg bandgaps or multiple bandgaps. For instance, Xiao et al.^7^ proposed a simple locally resonant continuous elastic system composed of a taut uniform string and periodically connected spring-mass resonators, clearly demonstrating the coexistence of two types of bandgaps in the system, namely Bragg bandgaps and resonance bandgaps, and the coupling of the two bandgaps generates an ultra-wide coupling bandgap. In the field of metasurfaces, Tsilipakos et al.^8^ proposed a achromatic gradient metasurfaces that achieves broadband and dispersionless operation. Tsilipakos et al.^9^ also proposed a practical, achromatic microwave metasurface for delaying broadband pulses in reflection by fitting five sharply resonant meta-atoms in a subwavelength unit cell, achieving a total bandwidth nearly linearly proportional to the number of resonances.

Piezoelectric materials have also been introduced into the designs of locally resonant metamaterials for their reconfigurability^10–13^. Airoldi and Ruzzene^10^ found in their research that the equivalent stiffness of a piezoelectric beam shunted with resonant circuits exhibits resonance characteristics near the resonance frequency. They proposed that such piezoelectric beams can be regarded as metamaterials, namely piezoelectric metamaterials. Sugino et al.^11^ studied one-dimensional locally resonant piezoelectric structures with segmented electrodes under transverse vibration and found that the formation of bandgaps is related to the resonance frequency in the shunting circuit and the electromechanical coupling coefficient of the piezoelectric patches. Yi et al.^12^ demonstrated through the analysis of the dynamic properties of piezoelectric metamaterial beams that bandgaps are generated by negative bending stiffness. Similar to passive locally resonant metamaterials, piezoelectric metamaterials also need to address the issue of bandgap widening. For example, Zhu et al.^14^ experimentally investigated the dynamic behavior of adaptive piezoelectric metamaterials with negative capacitance and obtained adjustable bandgaps. Sugino et al.^15^ proposed a hybrid unit structure made of piezoelectric laminates with segmented electrode pairs and additional mechanical resonators, which can simultaneously exhibit locally resonant bandgaps based on dynamic mass and stiffness. The combination of these two types of bandgaps enhances the overall bandgap. Jian et al.^16^ introduced a novel gradient piezoelectric metamaterial beam with parallel resonant circuits, employing a graded strategy for electrode pairs' spatial variation. With proper selection of resistors, the gradient piezoelectric metamaterial beam achieved theoretically the widest attenuation region. Celli et al.^17^ proposed that the heterogeneity of resonator properties and the interaction between spatial disorder could lead to the widening of the system's filtering effect, namely widening of the bandgaps, compared to more traditional concepts of spatially ordered rainbow materials. In order to enhance the practical performance of metamaterials, reduce the required number of unit cells, and additional mass, Airoldi and Ruzzene^18^ achieved multi-frequency resonance in piezoelectric metamaterials beams and simultaneously generated multiple bandgaps by introducing multi-frequency resonant circuits. Furthermore, Yi et al.^19^ designed a transfer function to achieve multi-resonance in digital circuits, realizing multi-resonant piezoelectric metamaterials based on self-inductive sensing piezoelectric patches and digital circuits. However, approaches for widening bandgaps in piezoelectric metamaterials are still somewhat limited.

Optimization methods have brought an avenue for the widening of the metamaterial’s bandgap. Common optimization schemes include topological optimization and parameter optimization^20–25^. Regarding passive elastic metamaterials, there is a considerable amount of research, for instance, Wu et al.^22^ proposed a genetic algorithm approach to design non-periodic unit structures, achieving broadband wave attenuation. Meng et al.^23^ established a theoretical model for connecting multiple local resonance bandgaps in two-phase composite materials with damping and obtained ultra-wide bandgaps through the proposed optimization procedure. Chen et al.^24^ employed an enhanced genetic algorithm to design square lattice structures, achieving wide and multiple bandgaps in the low-frequency range through optimized filler material distribution. For piezoelectric metamaterial, Jian et al.^25^ introduced a non-uniform piezoelectric metamaterial beam, where the shunting circuit parameters were optimized using an adaptive genetic algorithm to tailor the vibration attenuation region. Gao and Wang^26^ proposed a genetic algorithm to optimize the circuit parameters of LR parallel circuits in each unit of the metamaterial beam, which can couple multiple local resonance bandgaps to Bragg bandgaps to widen the frequency range of vibration suppression. In general, it remains an unresolved issue to widen bandgaps and achieve broadband vibration suppression in piezoelectric metamaterial plate through optimization methods.

This paper proposes a method to widen the bandgaps of multi-resonant piezoelectric metamaterials based on frequency merging phenomena. Firstly, a theoretical model of a piezo-metamaterial plate is obtained based on the effective medium theory, and criteria for determining the bandgap range in the metamaterial are provided by considering scenarios with and without damping. Subsequently, the bandgap merging phenomena between separated multi-bandgaps are analyzed. Then, based on the genetic algorithm and considering the bandgap merging phenomena, an optimization design method for widening the bandgaps is proposed. The bandgap widening effects by using the optimization method are studied separately for transfer functions with and without damping. Finally, numerical simulations are conducted to verify the effectiveness of widening bandgaps in the metamaterials.

## Materials and methods

### Multi-resonant piezoelectric metamaterial

#### Metamaterial plate physical model

**Figure 1:**
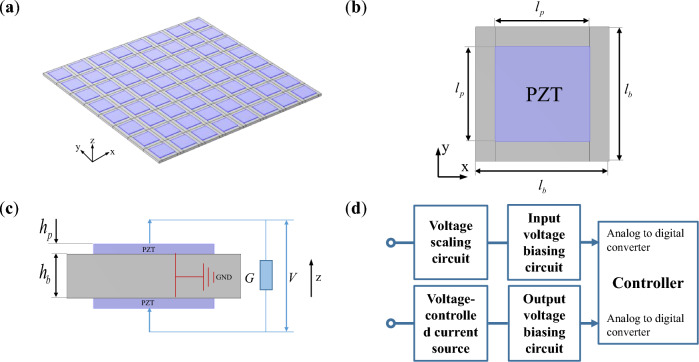
Sketch of the designed piezoelectric metamaterial plate: (**a**) metamaterial plate; (**b**) top view of unit; (**c**) side view of unit; (**d**) external digital circuit of unit^27^.

Figure 1 depicts a schematic diagram of the piezoelectric metamaterials plate and its unit. Each unit consists of a base plate, two piezoelectric patches attached to the upper and lower surfaces of the plate structure respectively, and digital synthesized impedance circuit connected to them. The metamaterial plate is composed of units arranged periodically in the plane. The polarization direction of the piezoelectric patches is aligned with the z-axis, and the surface electrode in contact with the plate is grounded. The digital synthesized impedance circuit includes voltage scaling circuit, input voltage biasing circuit, controller, output voltage biasing circuit, and voltage-controlled current source^27^, the overall effect of the circuit can be represented by the transfer function *G*. The material parameters and geometric dimensions of the base plate and piezoelectric patches in a metamaterial unit are shown in Table 1.

**Table 1:** Geometric and material parameters.

	Plate	Piezoelectric patches
Material	Aluminum	PZT-5H
Length (mm)	33.6	30.1
Width (mm)	33.6	30.1
Thickness (mm)	4	1
Young’s modulus (GPa)	70	58.8
Density (kg/m^3^)	2700	7700
Coupling constant (C/N)	–	− 1.7e−10
Relative permittivity under constant stress	–	1800

#### Bandgap determination criterion

Firstly, the equivalent parameters of the piezoelectric patch with shunting circuits are studied. The piezoelectric patch can be considered as isotropic thin plate. Based on the plane stress assumption and the sub-wavelength assumption, their equivalent in-plane Young's modulus and in-plane Poisson's ratio are^28^:

1 $$E_{p} = E_{p}^{sc} \frac{{G + sC_{p}^{T} }}{{G + sC_{p}^{T} \left( {1 - k_{31}^{2} } \right)}}$$

2 $$\nu_{p} = \nu_{p}^{sc} \frac{{G + sC_{p}^{T} \left( {1 + k_{31}^{2} /\nu_{p}^{sc} } \right)}}{{G + sC_{p}^{T} \left( {1 - k_{31}^{2} } \right)}}$$

where $E_{p}^{sc}$ and $\nu_{p}^{sc}$ are the in-plane Young's modulus and Poisson's ratio of the piezoelectric patch in the short-circuit state respectively, $k_{31} = d_{31} \sqrt {E_{p}^{sc} /\varepsilon_{3}^{\sigma } }$ is the piezoelectric coupling coefficient, $C_{p}^{T} = A_{p} \varepsilon_{3}^{\sigma } /h_{p}$ is the intrinsic capacitance of the piezoelectric patch under steady stress, $C_{p}^{s} = C_{p}^{T} \left( {1 - k_{31}^{2} } \right)$ is the intrinsic capacitance of the piezoelectric patch under steady strain, $s$ is the Laplace parameter, $d_{31}$ is the piezoelectric coupling constant of the patch, $\varepsilon_{3}^{\sigma }$ is the dielectric constant under steady stress, $A_{p}$ is the area of the patch, and $h_{p}$ is the thickness.

In the unit, according to classical laminated plate theory^29^, the equivalent bending stiffness of the laminated plate covered by the piezoelectric patch can be expressed as:

3 $$D_{A} = D_{b} + \frac{{E_{p} }}{{12\left( {1 - \nu_{p}^{2} } \right)}}\left[ {\left( {h_{b} + 2h_{p} } \right)^{3} - h_{b}^{3} } \right]$$

where $D_{b} = E_{b} h_{b}^{3} /12\left( {1 - \nu_{b}^{2} } \right)$ is the bending stiffness of the elastic plate, $E_{b}$ and $\nu_{b}$ are the Young's modulus and Poisson's ratio of the elastic plate, respectively.

Due to the different dimensions of the piezoelectric patch and the elastic plate, the equivalent parameters of the entire unit are calculated using the effective medium method^28^:

4 $$D_{eff} = \frac{{D_{b} \left[ {D_{b} + D_{p}^{sc} + D_{p}^{sc} \frac{{sC_{p}^{s} k_{31}^{2} \left( {1 + \nu_{p}^{sc} } \right)}}{{\left( {1 - k_{31}^{2} } \right)\left( {1 - \nu_{p}^{sc} } \right)\left( {G + sC_{p}^{s} } \right) - sC_{p}^{s} k_{31}^{2} \left( {1 + \nu_{p}^{sc} } \right)}}} \right]}}{{D_{b} + \left( {1 - \chi } \right)\left[ {D_{p}^{sc} + D_{p}^{sc} \frac{{sC_{p}^{s} k_{31}^{2} \left( {1 + \nu_{p}^{sc} } \right)}}{{\left( {1 - k_{31}^{2} } \right)\left( {1 - \nu_{p}^{sc} } \right)\left( {G + sC_{p}^{s} } \right) - sC_{p}^{s} k_{31}^{2} \left( {1 + \nu_{p}^{sc} } \right)}}} \right]}}$$

5 $$\rho_{eff} = \chi \left( {\rho_{b} h_{b} + 2\rho_{p} h_{p} } \right) + \left( {1 - \chi } \right)\rho_{b} h_{b}$$

where $\chi = l_{p}^{2} /l_{b}^{2}$ is the coverage ratio of the piezoelectric patch in the unit, $D_{p}^{sc} = E_{p}^{sc} \left[ {\left( {h_{b} + 2h_{p} } \right)^{3} - h_{b}^{3} } \right]/12\left( {1 - \nu_{p}^{sc2} } \right)$ is the equivalent bending stiffness of the laminated plate covered by the piezoelectric patch when the piezoelectric patch is short-circuited, $\rho_{b}$ and $\rho_{p}$ are respectively the densities of the elastic plate and the piezoelectric patch. The transfer function *G* is the multi-resonant transfer function designed by the authors in^19^. It makes the equivalent Young's modulus $E_{p}$ of the piezoelectric patch resonate at multiple frequencies, and multiple bandgaps are generated in the metamaterial. The expression of the transfer function is:

6 $$G\left( s \right) = s\gamma C_{p3}^{T} \frac{{\prod\limits_{i = 1}^{n} {\left( {s^{2} + 2\beta_{i} \omega_{p,i} s + \omega_{p,i}^{2} } \right) - \prod\limits_{i = 1}^{n} {\left( {s^{2} + 2\beta_{i} \omega_{z,i} s + \omega_{z,i}^{2} } \right)} } }}{{\frac{1}{{1 - k_{31}^{2} }}\prod\limits_{i = 1}^{n} {\left( {s^{2} + 2\beta_{i} \omega_{z,i} s + \omega_{z,i}^{2} } \right) - } \prod\limits_{i = 1}^{n} {\left( {s^{2} + 2\beta_{i} \omega_{p,i} s + \omega_{p,i}^{2} } \right)} }}$$

$\beta_{i}$, $\omega_{z,i}$, and $\omega_{p,i}$ represents the damping, zeros, and poles in the transfer function *G* respectively, and they correspond one-to-one, with their quantity determined by the number of poles *n.* The poles can be expressed as $\omega_{p,i} = 2\pi f_{i}$, $f_{i}$ corresponding to the resonance frequencies of the poles. Then, *n* bandgaps at the *n* resonance frequencies corresponding to the poles are produced at the metamaterial. The transfer function should satisfy the Nyquist stability criterion, i.e., its poles should all be located in the left half-plane of the complex plane, and the relationship between poles and zeros is obtained^19^:

7 $$\omega_{z,i} < \omega_{p,i} < \left( {\frac{1}{{1 - k_{31}^{2} }}} \right)^{\frac{1}{2n}} \omega_{z,i} \, ,i = 1,2,...,n$$

**Figure 2:**
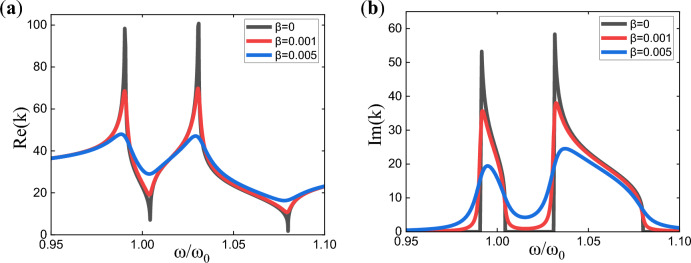
Dispersion curves corresponding to a two-pole transfer function with different level of damping.

The frequency dispersion relationship of the metamaterial can be expressed by the equivalent properties of it. Here, we consider the dispersion relation in the *x*-direction of the metamaterial. The dispersion relation of the metamaterial with two poles configured in the transfer function is shown in Fig. 2, where Fig. 2a and b respectively depict the variation curves of the real and imaginary parts of the wavenumber with frequency. The dispersion relation is described using complex wavenumbers:

8 $$\it k = Re \left( k \right) + jIm \left( k \right) = \sqrt {\omega \sqrt {\frac{{\rho_{eff} }}{{D_{{eff}} }}} }$$

Assume a harmonic wave solution $u = Ae^{j(kx - \omega t)}$, from Eq. (8), we can obtain:

9 $$u = Ae^{{ - {\text{Im}} \left( k \right)x}} e^{{j\left( {{\text{Re}} \left( k \right)x - \omega t} \right)}}$$

This indicates, the real part of the wavenumber represents the phase change of the wave, while the imaginary part of the wavenumber represents the attenuation of the amplitude in space.

Combining Eqs. (4), (5), and (8), it can be observed that when the material and geometric dimensions of the metamaterial are determined, the wavenumber value is only related to the transfer function *G*. According to the expression of the transfer function (6), the parameters affecting wavenumber are $n$, $\beta_{i}$, $\omega_{z,i}$, $\omega_{p,i}$. Pole $\omega_{p,i}$ and zero $\omega_{z,i}$ affect the location where the poles are generated, while damping $\beta_{i}$ directly affects the wave propagation effect.

In Fig. 2, there are three curves each for the real and imaginary parts of the wavenumber dispersion curves, with different damping configurations. In Fig. 2a, as the damping increases, the resonance effect of the real part of the wavenumber gradually weakens. In Fig. 2b, when the damping is zero, there is a clear range of zero values and non-zero values for the imaginary part of the wavenumber, where the non-zero range represents the bandgap range. And if the damping is non-zero, there is no longer a strict zero-value range for the imaginary part of the wavenumber. Therefore, in these cases, we define the regions where the imaginary part of the wavenumber ${\text{Im}} \left( k \right)$ is larger than a certain value *k*_*th*_ as bandgaps. We require that the vibration decreases by at least − 10 dB after passing through four unit cells. According to Eq. (9), $20\log \left( {\frac{{\left| {u_{2} } \right|}}{{\left| {u_{1} } \right|}}} \right) = 20\log \left( {e^{{ - {\text{Im}} (k) \cdot \left( {x_{2} - x_{1} } \right)}} } \right) = - 10$ (subscripts 1 and 2 represent two points in the direction of vibration transmission, and the distance between them is four unit lengths), which gives the imaginary part of the wavenumber to be greater than 8.5534. Therefore, we have chosen 10 as the bandgap criterion value here, i.e., *k*_*th*_ = 10. Results in Fig. 2 (and other references^19^) also demonstrate that, in terms of wave attenuation, damping has positive effect between the two poles but has negative effect at and near the vicinity of the two poles. Therefore, increasing damping blindly is not advisable. Instead, damping should be optimized to obtain the best attenuation characteristics.

#### Bandgap merging phenomenon

When multiple poles are configured in the multi-resonant transfer function, piezoelectric metamaterials can generate multiple bandgaps. It has been found in studies that there is a coupling interaction between these multiple bandgaps. When the bandgaps are close enough, they merge into one, and the merged bandgap is wider than the original multiple individual bandgaps^19^.

**Figure 3:**
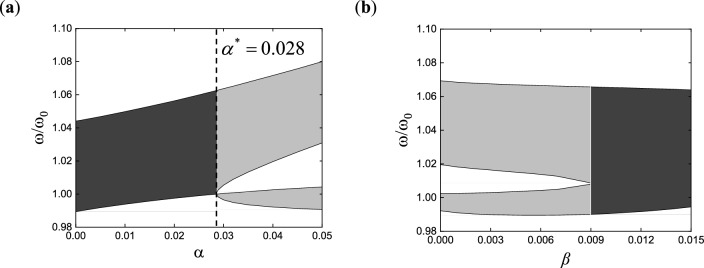
Bandgap changes with (**a**) pole spacing or (**b**) damping.

Taking the case of two poles configured in the transfer function as an example, the phenomenon of bandgap merging is illustrated in Fig. 3. When the damping in the transfer function is zero, the bandgap merging is dominated by the distance between poles, as shown in Fig. 3a. If the distance between poles is less than or equal to $\alpha^{*}$ = 0.028, two independent bandgaps merge, and the merged one is wider than the original two separate bandgaps. When there is damping in the transfer function, besides the influence of the distance between poles, the range of the bandgap is mainly affected by the damping magnitude, as shown in Fig. 3b. The distance between poles α in the transfer function is set to 0.04, and the equal damping corresponding to the two poles is taken as the independent variable, namely $\beta_{1} = \beta_{2} = \beta$. It can be observed that, without changing the distance between poles, as the damping increases, the widths of the two independent bandgaps increase, and bandgap merging occurs when the damping equaling a certain value. It should be noted that after bandgap merging, increasing the damping continuously leads to a decrease in the bandgap width. From Fig. 3, it can be concluded that widening the bandgap can be achieved by merging multiple bandgaps in multi-resonant piezoelectric metamaterials.

When the number of poles in the transfer function exceeds 2, the situation of bandgap merging becomes more complex. This paper considers using optimization algorithms to find the optimal effect of bandgap merging when configuring multiple poles in the transfer function.

### Optimization method

Due to the different methods of determining the bandgap range, it is necessary to discuss separately for $\beta_{i} = 0$ and $\beta_{i} > 0$ in the optimization calculation, with their corresponding bandgap criteria being Im(*k*) > 0 and Im(*k*) > *k*_*th*_, respectively.

The optimization aims to merge multiple independent bandgaps generated by multiple poles into a wider bandgap. Since the merged bandgap is wider than the individual bandgaps before merging, the optimization objective is to maximize the length of the frequency range of the widest bandgap among all the bandgaps in the multi-pole metamaterial. After determining the parameters of the transfer function, the imaginary part of the wavenumber values can be obtained from Formula (8). From these values, the frequency range of the bandgap is identified, and the left boundary frequency of the widest bandgap is denoted as $\omega_{1}$, while $\omega_{2}$ is for the right boundary. Therefore, the objective function for optimization is formulated as follows:

10 $$J = \max \left( {\omega_{2} - \omega_{1} } \right)$$

where the variable parameters are $n$, $\beta_{i}$, $\omega_{z,i}$, and $\omega_{p,i}$. To improve optimization efficiency, it is necessary to consider additional constraints to limit the design space.

In the case of two-pole transfer function, the bandgap merging in metamaterials occurs only when the separation between poles is less than a critical value $\alpha^{*}$. However, in situations involving multiple poles (n > 2), due to coupling effects^19^, the critical value for pairwise merging of bandgaps may be smaller than $\alpha^{*}$. Therefore, choosing $\alpha^{*}$ to limit the range of pole value search is rational.

For the cases with damping, the bandgap merging phenomenon depends on the value of $\beta$ and the distance between two poles. But, if the distance between two poles is too large, no matter what value the $\beta$ is chosen, the two bandgaps cannot be merged. Therefore, in our optimization procedure, to make the algorithm more efficient, we restrict the distance of two poles to be less than a certain value. In our studies, we choose $2\alpha^{*}$*,* and the results demonstrate that we can find all the optimized parameters using this value. In other studies with different material and geometry parameters, one may need to increase this value if the optimization algorithm fails. A metric coefficient *p* is introduced for $\alpha^{*}$, namely $p = 1$ when $\beta_{i} = 0$, and $p = 2$ when $\beta_{i} > 0$. When considering damping in the transfer function, an appropriate damping factor $\beta$ should be selected to adjust the bandgap effect. However, the damping coefficient should not be too large to avoid weakening the resonance effect of the shunt circuit. Here, the search range for *β* is chosen to be less than 0.1. The searching range of *β* must be enlarged if the algorithm fails to find the optimized parameters. The constraints on variables $\omega_{p,i}$ and $\beta_{i}$ limit the design space to:

11 $$\begin{gathered} \omega_{0} < \omega_{p,i} \le \left[ {1 + p \cdot \left( {i - 1} \right)\alpha^{*} } \right]\omega_{0} \, ,i = 1,...,n;p = 1,2 \\ 0 \le \beta_{i} \le 0.1 \, ,i = 1,...,n \\ \end{gathered}$$

where $\omega_{0}$ is the target angular frequency, $\omega_{0} = 2\pi f_{0}$, the metamaterial generates a bandgap at frequency *f*_*0*_ to suppress structural vibrations. During the optimization process, $\omega_{p,1}$ is fixed as the target frequency. For convenience in the optimization process, the pole values are normalized, by setting $\omega_{p,i}^{\prime } = \omega_{p,i} /\omega_{p,1}$.

**Figure 4:**
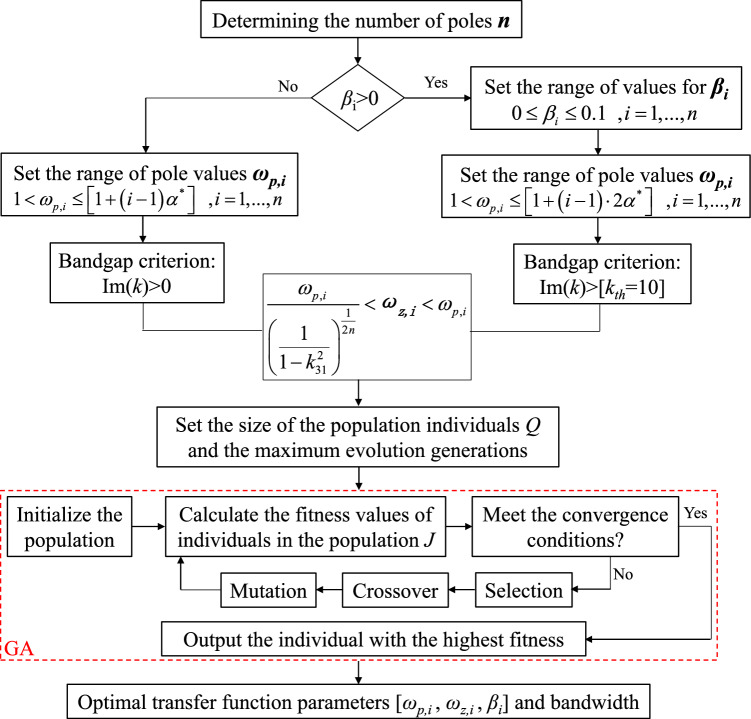
Flow chart of optimization algorithm to calculate optimal bandgap.

In this paper, genetic algorithms are utilized to solve optimization problems with a specified objective *J*. The principle of genetic algorithms mimics the natural evolutionary process, where individuals better adapted to the environment have higher chances of producing offspring for the next generation, gradually evolving towards superior solutions. The evolution process begins with a randomly generated initial population containing Q individuals, with each individual represented by a vector $\left[ {\omega_{p,2} , \ldots ,\omega_{p,n} ,\omega_{z,1} , \ldots ,\omega_{z,n} ,\beta_{1} , \ldots ,\beta_{i} } \right]$ (which becomes $\left[ {\omega_{p,2} , \ldots ,\omega_{p,n} ,\omega_{z,1} , \ldots ,\omega_{z,n} } \right]$ when damping terms are disregarded), where the parameters in the vector are subject to constraints (7) and (10). The optimization objective function (9) is directly employed as the fitness function to evaluate the quality of individuals. Based on the obtained fitness values, three operators—selection, crossover, and mutation—are applied to individuals in the population to generate better offspring. Subsequently, a new generation is produced, and the aforementioned process is repeated. Through successive iterations, when further improvements become unattainable, the optimal solution is obtained. Figure 4 illustrates the flowchart of the optimization algorithm design.

## Results and discussion

### The damping term in the transfer function is zero

**Figure 5:**
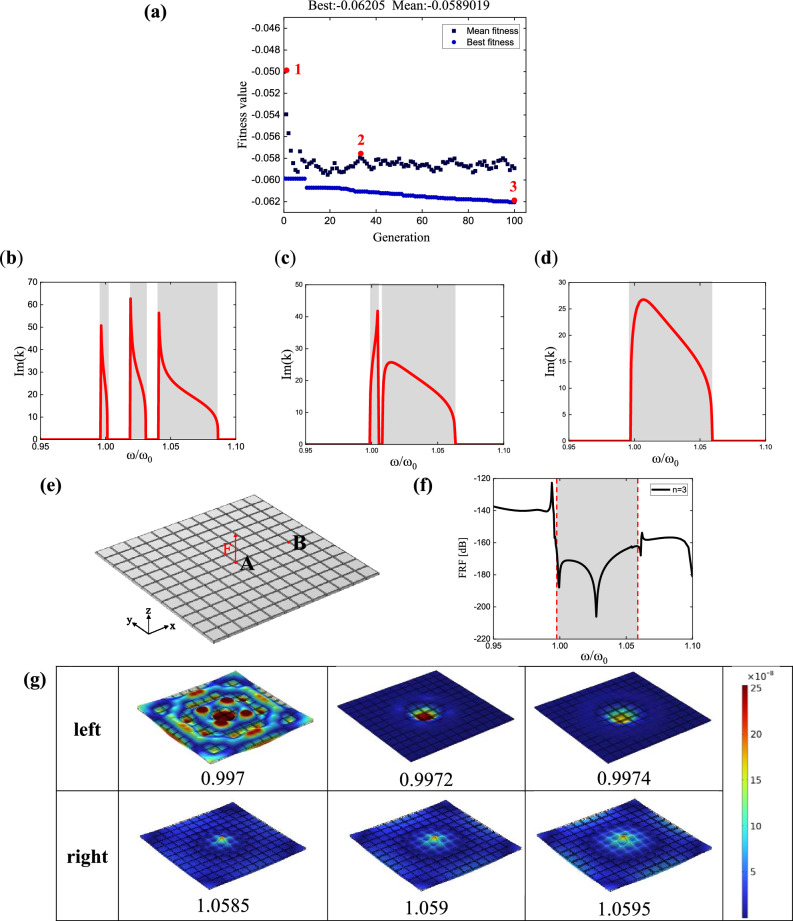
Bandgap merging results of the non-damping three-pole metamaterial: (**a**) convergence plot of the optimized fitness value evolution; (**b**–**d**) are the dispersion curves of the metamaterial, with transfer function parameters taken from points 1 (The pole values are $\omega_{p} = [1,1.028,1.056]$), 2 (The pole values are $\omega_{p} = [1,1.01,1.026]$), and 3 [Parameters are shown in Eq. (12)] in (**a**), respectively; (**e**) is the metamaterial plate model used for simulation; (**f**) is the response curve at point B obtained from the simulation; (**g**) shows the mode shapes of the metamaterial plate, with three frequencies taken on each side of the theoretical bandgap, the numbers represent the normalized frequency, and the color is value of the longitudinal displacement |*w*|.

The optimization strategy above is used to optimize the case of $$\beta_{i} = 0$$. In this case, the number of variables is $$\left( {2n - 1} \right)$$. Taking the case with three poles, the constraint on variable $$\omega_{p,i}$$ is set to $$1 < \omega_{p,i} \le \left[ {1 + \left( {i - 1} \right) \cdot 0.028} \right]$$$$\left( {i = 2,3} \right)$$. The population size is set to Q = 50. Figure 5a illustrates the evolution of fitness values during the optimization process. Here are the optimal parameter values obtained through optimization when the number of poles *n* is 3:

12 $$\begin{gathered} \omega_{p} = [1,1.00314,1.02219] \hfill \\ \omega_{z} = [0.98137,0.98445,1.00315] \hfill \\ \end{gathered}$$

To clearly illustrate the evolution of bandgap merging, the dispersion curves of metamaterial unit cells are used to represent the process, with parameters chosen from the initial generation, intermediate generations, and optimal results, as shown in Fig. 5b–d. It can be observed that in the initial generation, Fig. 5b, there are three independent bandgaps, while in the optimal result, Fig. 5d, only one bandgap remains, formed by the merging of the three independent bandgaps, and its width is greater than that of the individual bandgaps.

As is well known, genetic algorithm is time-consuming, with much of the computational resources spent on repeatedly calculating fitness values. Therefore, the convergence time of the algorithm strongly depends on the population size ($\left( {2n - 1} \right) \times Q$) and the complexity of the fitness function. The larger the number of poles *n*, the more time is required for computation. Therefore, here we first consider cases with 10 poles for calculation. The optimization results are shown in Table 2. It can be seen that the width of the merged bandgap after combining multiple poles is slightly wider than that of a single bandgap.

**Table 2:** Bandgap width under multi-pole bandgap merging ($\beta_{i} = 0$).

n	1	2	3	4	5	6	7	8	9	10
Bandgap	0.06	0.0626	0.062	0.0646	0.0627	0.0635	0.0607	0.0633	0.0625	0.062

The metamaterial generates bandgaps in the structure through locally resonant units, which achieve structural vibration control by blocking the propagation of elastic waves within the corresponding frequency range. Then, the elastic wave transmission isolation characteristic of the bandgap is applied, and the optimized metamaterial parameters are selected to simulate the vibration transmission isolation effect in the metamaterial plate in the frequency domain, so as to verify the optimization results.

A simulation model of a piezoelectric metamaterials plate is established in COMSOL6.0, as shown in Fig. 5e. The plate is composed of a 12 × 12 array of metamaterial units illustrated in Fig. 1b, with material parameters and geometric dimensions listed in Table 1. In the metamaterial plate, piezoelectric patches are attached to the upper and lower surfaces of the substrate plate. Add Solid Mechanics and Electrostatics as two physical fields, and couple them to form the piezoelectric effect through multi-physics coupling. Apply free boundaries to the metamaterial plate in the solid mechanics module. In the charge module, ground the surface of the piezoelectric element adhered to the substrate plate. Adding a weak boundary contribution on the outer surface of the piezoelectric patch to connect the circuit with compliance G to the piezoelectric patch in the COMSOL model. Weak contributions are functionalities used internally in COMSOL for applying built-in domains and boundary conditions. Here, the specific weak expression is given by $\frac{test\left( V \right) \cdot V}{{iA_{p} \omega }} \cdot G$. It represents the boundary condition of adding charges on the outer surface of the piezoelectric patch. Here, ${A}_{p}$ is the area of the surface of a piezo-patch, *G* is the transfer function in Eq. (6), *V* is the voltage of the DOF, *test*(·) is an embedded function in COMSOL.

The central position of the simulated plate model is denoted as point A, which a unit harmonic point load F is applied to simulate the disturbance applied to the structure, within the solid mechanics module. Point B is marked to the right of point A in the x-direction and is used to measure the vertical displacement response |*w*| in the frequency domain due to the disturbance source, thereby verifying the effectiveness of vibration transmission isolation. The frequency response function can be used to characterize the transmission effect of vibration from the source to the measurement position in the frequency domain. Dividing the vibration displacement response by the amplitude of the excitation force yields the frequency response function at point B. Once the model is configured, select the Frequency Domain Study in COMSOL. Add computation frequency points for analysis, and examine the simulation results to observe the effects.

Taking the example of three-pole, the theoretical calculation of the normalized bandgap range is $\left[ {0.9971,1.0591} \right]$. Figure 5f shows the frequency response curve of point B under the effect of the transfer function with three-pole (solid line), while the shaded region represents the theoretical bandgap range, and the dashed lines denote the normalized frequency values of the simulated bandgap boundaries. The displacement response expresses the vertical displacement |*w*| of the plate caused by its bending stiffness. Three frequencies are selected near the theoretical bandgap boundaries to observe the corresponding vibrational modes, as shown in Fig. 5g. From Fig. 5f, it can be observed that the frequency response function (FRF) of the numerical results exhibits a trough within the theoretical bandgap range, indicating a significant reduction in displacement at the measurement point. It indicates that this interval represents the bandgap range, as the bandgap blocks the propagation of elastic waves within the plate, preventing the vibration generated by the central excitation of the plate from propagating to the measurement point in the far field. The vibrational mode plots in Fig. 5g provide a more intuitive demonstration of the bandgap effect. At 0.997, the metamaterial plate undergoes significant deformation, while at 0.9972, the deformation is localized at the center of the plate, indicating that the frequency is within the bandgap, and the bandgap blocks the elastic wave propagation generated by excitation. The theoretical and numerical bandgap boundary frequencies are in good agreement, with the left boundary frequency matching well. At the right boundary, beyond the theoretical bandgap boundary frequency of 1.059, the deformation of the metamaterial plate increases, suggesting that 1.059 can be considered as the right boundary frequency of the bandgap. Therefore, the theoretically calculated bandgap range is in good agreement with the numerical simulation results.

**Figure 6:**
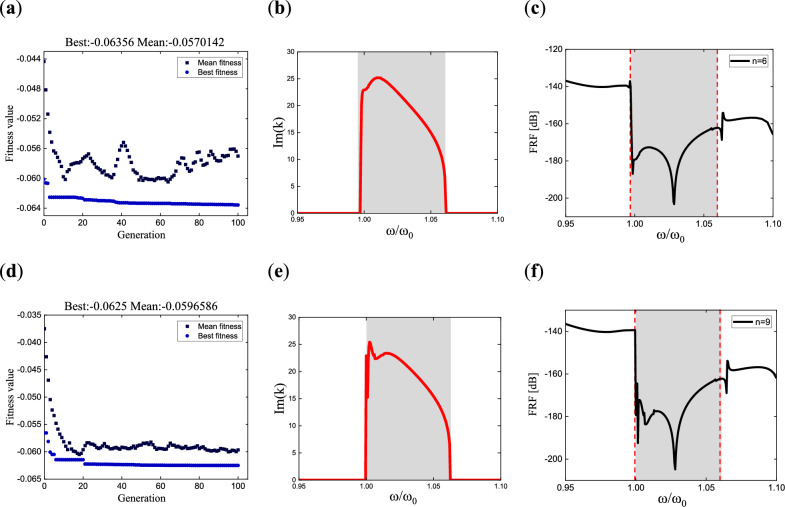
Fitness value evolution, dispersion curve, and frequency response curve of the non- damping multi-pole piezoelectric metamaterials: (**a**–**c**) correspond to the six-pole metamaterial with the pole values of $\omega_{p} = [1,1.000032,1.0001,1.00317,1.01268,1.02224]$, while (**d**–**f**) correspond to the nine-pole metamaterial with the pole values of $\omega_{p} = [1,1.0004,1.00165,1.0054,1.0057,1.006,1.012,1.0124,1.0192]$.

In addition, the results of $n = 6,9$ are presented in Fig. 6, with three plots corresponding to each case. These plots depict the evolution of fitness values during the optimization process, the dispersion curves of the metamaterial under the optimal parameters, and the frequency response curves. In the frequency response curves (c) and (f), the theoretical bandgap range is also represented by shaded regions, while the simulated bandgap boundaries are depicted by dashed lines with normalized frequency values. The bandgap determination method is the same as that of the three-pole transfer function. For $n = 6$, the theoretical bandgap range is $\left[ {0.9975,1.061} \right]$, while the simulated bandgap is $\left[ {0.9975,1.061} \right]$. For $n = 9$, the theoretical bandgap range is $\left[ {1.0,1.0624} \right]$, and the simulated bandgap is $\left[ {1.0,1.062} \right]$. The results show that the theoretical and simulated bandgap ranges are in good agreement.

The above analysis results show that the merging of multiple bandgaps can indeed widen the bandgap, but the effect is not very significant. Then, the reason why this method cannot significantly widen the bandgap is analyzed in detail.

The inequality (7) provides a constraint relationship between zeros and poles, which can be reformulated as follows:

13 $$\frac{{\omega_{z,i} }}{{\omega_{p,i} }} \in \left( {\frac{1}{{\left( {\frac{1}{{1 - k_{31}^{2} }}} \right)^{\frac{1}{2n}} }},1} \right)$$

**Figure 7:**
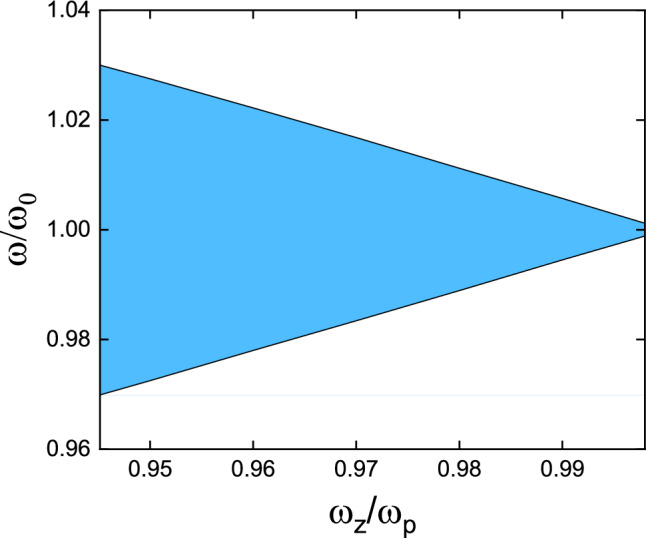
Bandgap width changes with the zero-pole ratio.

The range of value for the ratio in Eq. (13) narrows as the number of poles *n* increases. When considering no damping, the single-pole transfer function has only two variables: pole $\omega_{p}$ and zero $\omega_{z}$. Analyzing the variation of bandgap width with the zero-pole ratio $\omega_{z} /\omega_{p}$, as shown in Fig. 7, it can be observed that the bandgap width decreases monotonically with the increase in the zero-pole ratio. Equation (13) indicates that as the number of poles *n* increases, the zero-pole ratio increases compared to the left boundary of the value range. This implies that the range of the zero-pole ratio becomes smaller and smaller. As shown in Fig. 7, the corresponding bandgap width also becomes narrower. The width of each bandgap in metamaterials decreases as the number of poles n increases. This also leads to the fact that increasing *n* does not significantly increase the width of the merged bandgap.

### Taking into account the damping effect in the transfer function

Using the optimization strategy mentioned above, optimization calculations are performed for the case of $$\beta_{i} > 0$$. The number of variables is $$\left( {3n - 1} \right)$$. Taking *n* = 3 as an example, the constraints for variable $$\omega_{p,i}$$ are set as $$1 < \omega_{p,i} \le \left[ {1 + \left( {i - 1} \right) \cdot 0.048} \right]$$$$\left( {i = 2,3} \right)$$, and the constraints for variable $$\beta_{i}$$ are set as $$0 < \beta_{i} < 0.1$$. The number of individuals is set to Q = 100. Below are the optimal parameter values obtained from the optimization when *n* = 3:

14 $$\begin{gathered} \omega_{p} = [1,1.030475,1.072786] \hfill \\ \omega_{z} = \left[ {0.98137,1.011277,1.0528} \right] \hfill \\ \beta = [0.006243,0.015848,0.016432] \hfill \\ \end{gathered}$$

**Figure 8:**
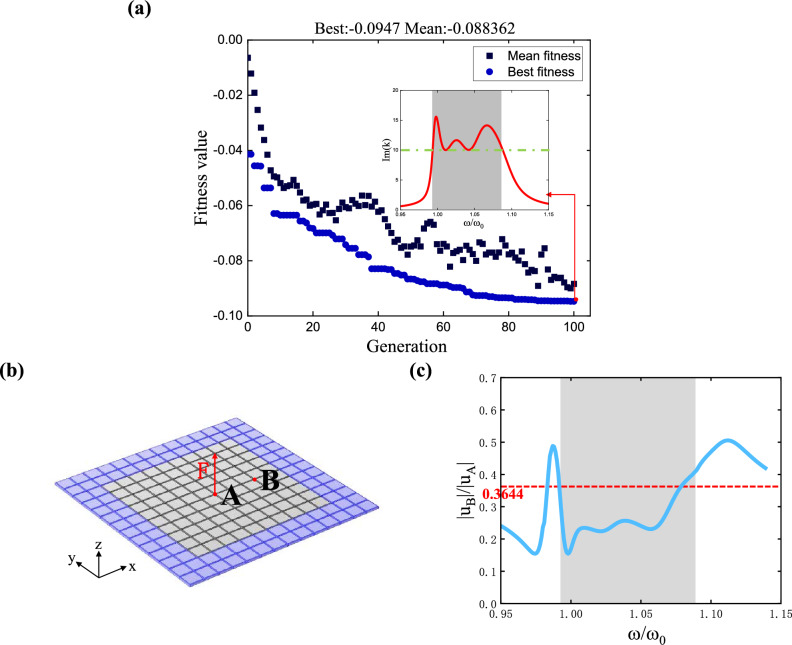
Bandgap merging results of the damped three-pole metamaterial: (**a**) theoretical calculation results: the main plot shows the convergence of the optimized fitness value evolution, and the inset displays the dispersion curve under optimized results with parameters given in Eq. (14); (**b**) the metamaterial plate model used for simulation with damping; (**c**) the ratio of points B to A obtained from the simulation as a function of frequency.

Figure 8a depicts the evolution of fitness values during optimization and the corresponding dispersion curves for the final optimized parameters. In the subplot, the solid line represents the dispersion curve, while the dashed line indicates *k*_*th*_ = 10.

Here, the case with ten poles is also considered for calculation. The optimization results are shown in Table 3:

**Table 3:** Bandgap width under multi-pole bandgap merging ($\beta_{i} > 0$).

n	1	2	3	4	5	6	7	8	9	10
Bandgap	0.0602	0.0808	0.0947	0.1041	0.1087	0.1132	0.1164	0.119	0.1193	0.1195

Comparing optimization results with and without damping. It can be observed that when considering the damping effect, the width of the merged bandgap of the multi-pole metamaterial increases with the number of poles *n*, but the rate of increase gradually slows down as *n* increases. Under the same *n*, the presence of damping significantly widens the bandgap. For instance, in the case of a damped ten-pole metamaterial, the bandgap is 200% wider than the without damping bandgap.

Similarly, the above optimization results are verified through simulations in COMSOL6.0. Due to the presence of damping, it is not possible to directly determine the bandgap position from the mode diagram of the metamaterial plate. Therefore, we constrain the frequency band where the imaginary part of the wavenumber is greater than 10 to indicate the bandgap. Here, the bandgap position is verified through the wavenumber values obtained from simulation results.

Using the same approach as the previous section, the simulation model is established. However, in this case, wavenumber calculation is required to consider the propagation of elastic waves. Therefore, perfectly matched layers are added around the metamaterial plate to absorb reflected waves, as indicated by the dark region surrounding the plate in Fig. 8b. Specifically, establishing a fictitious domain in the Definition, adding a perfect matching layer, and selecting the domain as all substrates and piezoelectric patches in the dark-colored regions. The metamaterial plate, excluding the perfectly matched layers, consists of a 10 × 10 array of metamaterial units, with excitation applied at point A and measurement taken at point B.

In the simulation model, a unit harmonic load is applied at point A in the center of the metamaterial plate, generating elastic waves in it. From the wave solution in Eq. (9), the amplitude ratio between point B and point A can be obtained as:

15 $$\frac{{\left| {u_{{\text{B}}} } \right|}}{{\left| {u_{{\text{A}}} } \right|}} = \frac{{Ae^{{ - {\text{Im}} \left( k \right)x_{B} }} }}{{Ae^{{ - {\text{Im}} \left( k \right)x_{A} }} }} = e^{{{\text{Im}} \left( k \right)(x_{A} - x_{B} )}} = e^{{ - 3l_{b} \cdot {\text{Im}} \left( k \right)}}$$

That is, ${\text{Im}} \left( k \right) = - \ln \left( {\frac{{\left| {u_{{\text{B}}} } \right|}}{{\left| {u_{{\text{A}}} } \right|}}} \right)/\left( {3l_{b} } \right) > 10$, where $l_{b}$ represents the geometric dimensions of the base structure in the metamaterial unit. This leads to the requirements for the amplitude ratio between point B and point A corresponding to the bandgap range:

16 $$\frac{{\left| {u_{{\text{B}}} } \right|}}{{\left| {u_{{\text{A}}} } \right|}} < e^{ - 3lb \cdot 10} = 0.3644$$

In the COMSOL Definition module, set up two point integrations (intop), selecting points A and B. Define the variable $V1 = \left| {u_{{\text{B}}} } \right|/\left| {u_{{\text{A}}} } \right|$ to compute the displacement ratio between the two points. After conducting frequency domain analysis, we can plot the curve in the results.

Figure 8c investigates the metamaterial with a transfer function featuring three-pole. The solid line represents the variation of the displacement amplitude ratio between point B and point A with frequency in the simulation results, while the dashed line indicates the required ratio. It can be observed that the range satisfying the above ratio requirement is $[0.993,1.083]$, and the theoretically obtained bandgap is $[0.9934,1.0881]$ (Indicated in the figure by shaded regions), the left boundaries of both are essentially the same, while the simulated results on the right boundary are slightly smaller. However, the error between the simulated bandgap width and the theoretical bandgap width is less than 5%, so the simulated results can be considered reasonable.

**Figure 9:**
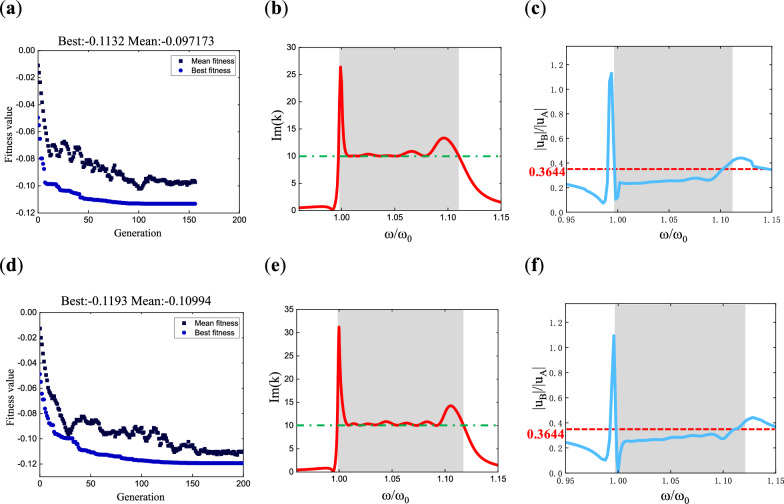
Fitness value evolution, dispersion curve, and ratio curve of points B to A for damped multi-pole piezoelectric metamaterials: (**a**–**c**) correspond to the six-pole metamaterial with the pole values of $\omega_{p} = [1,1.01094,1.02656,1.04598,1.06835,1.09652]$ and the damping values of $\beta = [0.00046,0.010475,0.01353,0.0164,0.0148,0.011399]$; (**d**–**f**) correspond to the nine-pole metamaterial with the pole values of $\omega_{p} = [1,1.0017,1.0142,1.0273,1.0354,1.0457,1.0649,1.0843,1.105]$ and the damping values of $\beta = [0.0011,0.0045,0.008,0.01,0.0217,0.0114,0.0117,0.011,0.0074]$.

Additionally, the results for *n* = 6 and *n* = 9 are presented in Fig. 9, including the evolution of fitness values during optimization, the dispersion curves of the metamaterial obtained under the optimal parameters, and the variation of the amplitude ratio between two points in the corresponding simulation results. Dashed lines are used in the amplitude ratio curves (c) and (f) to indicate the bandgap ranges, applying the method described by Eq. (16) for determination. The theoretical bandgap range for *n* = 6 is $\left[ {0.9971,1.1103} \right]$, while the simulated bandgap is $\left[ {0.998,1.105} \right]$. Similarly, for *n* = 9, the theoretical bandgap range is $\left[ {0.9982,1.1175} \right]$, and the simulated bandgap is $\left[ {0.999,1.113} \right]$. The left bandgaps in both cases are essentially the same, while the simulated results for the right bandgap are slightly smaller but within the acceptable error range. Therefore, the simulated results can be considered reasonable.

**Figure 10:**
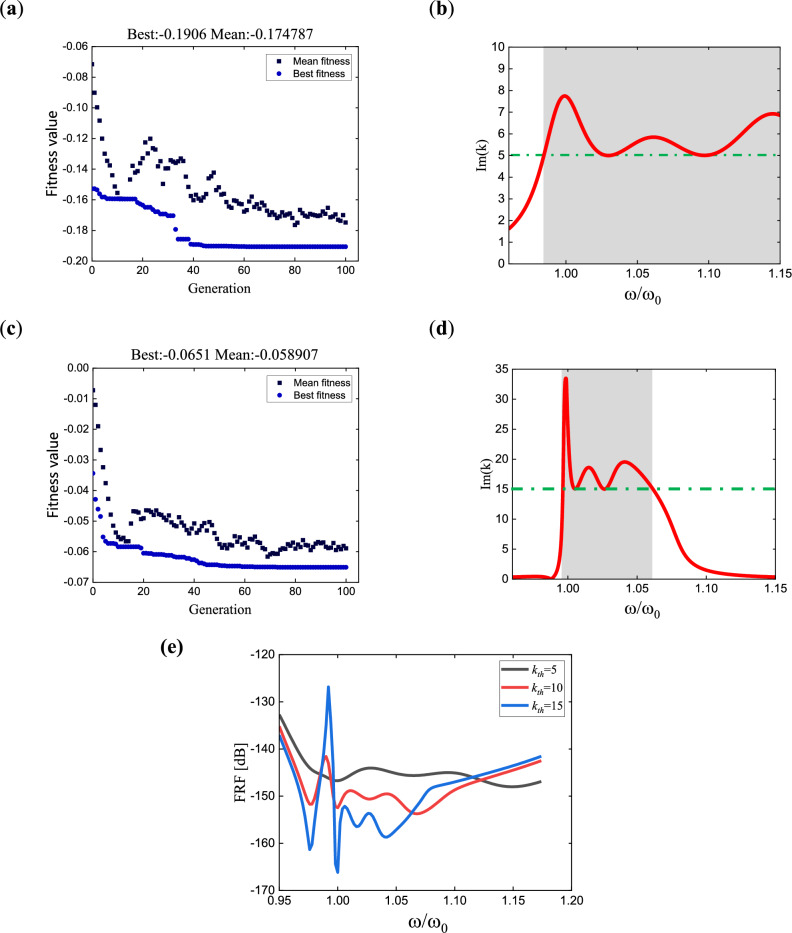
Detailed explanation of the meaning of *k*_*th*_, using the three-pole metamaterial as an example: (**a**, **b**) show the convergence plot of the optimized fitness value and the dispersion curve of the metamaterial for *k*_*th*_ = 5, respectively; (**c**, **d**) show the results for *k*_*th*_ = 15; (**e**) summarizes the displacement responses under optimized results for *k*_*th*_ = 5, 10, and 15.

In the previous analysis, the frequency range where the imaginary part of the wave vector is greater than 10 is defined as the bandgap. Here, we discuss the influence of wavenumber selection on the bandgap range for *n* = 3 by considering other wavenumber values. Optimization calculations are performed for *k*_*th*_ = 5 and *k*_*th*_ = 15, and the results are shown in Fig. 10. Combining with Fig. 8, it can be observed that when a smaller *k*_*th*_ is chosen for optimization, a wider bandgap range is obtained; whereas, with a larger* k*_*th*_, the resulting bandgap range is narrower. In real applications, *k*_*th*_ is determined according to the desired wave attenuation level. For example, if we want at least 50% reduce of the amplitude of a wave when it travels from point A to B, we can find the value of *k*_*th*_ by $k_{th} \ge - \ln \left( {\frac{{\left| {u_{{\text{B}}} } \right|}}{{\left| {u_{{\text{A}}} } \right|}}} \right)/\left( {l_{AB} } \right) = - \ln \left( {0.5} \right)/\left( {l_{AB} } \right)$, $l_{AB}$ is the distance between point A and B.

The optimization results obtained under three different wavenumber constraints are compared through simulations in COMSOL. Using the infinite model depicted in Fig. 8b, a unit point load is applied at the center of the metamaterial plate, and the frequency responses measured at the same position on the periphery are shown in Fig. 10e. The imaginary part of the wavenumber represents the decay of the amplitude in space. From the figure, it can be observed that within the narrowest band corresponding to *k*_*th*_ = 15 (i.e., the range [0.9964, 1.0615]), the response curve represented by the bottom blue line, which corresponds to the constraint of *k*_*th*_ = 15, exhibits the smallest response amplitude and the fastest decay. Conversely, the response curve for *k*_*th*_ = 5, indicating the smallest wavenumber constraint, exhibits the largest response amplitude and the slowest decay. However, from a wider frequency range, the response curve for *k*_*th*_ = 5 shows attenuation over a larger range, while the response curve for *k*_*th*_ = 15 has the smallest impact range. Reducing the constraint on the wavenumber can increase the range of attenuation, but it also weakens the attenuation effect.

## Conclusion

This paper presents a method for widening the bandgap of multi-resonant piezoelectric metamaterials. The equivalent properties of the metamaterial plate are derived, and the dispersion relation represented by complex wavenumbers is obtained. By analyzing the influence of damping effects on the dispersion relation in the transfer function, criteria for determining the bandgap range with and without damping in the transfer function are provided. By discussing the phenomenon of bandgap merging in metamaterials, an optimization design method for widening the bandgap in metamaterials based on genetic algorithms is proposed. Optimization designs are conducted for both with and without damping multi-pole metamaterials, and in both cases, the optimized bandgaps are wider than the previous independent ones. Without damping, the bandgap can be slightly widened, while with damping, the bandgap frequency range can be significantly expanded by over 200% through bandgap merging. This validates the effectiveness of the method for widening the bandgap in piezoelectric metamaterials. The proposed method for widening the bandgap in metamaterials can achieve broad vibration suppression capabilities at the target frequency and can actively tune the frequency range of vibration suppression.

## Data Availability

The datasets generated during and/or analyzed during the current study are available from the corresponding author on reasonable request.
